# Population-based study of laparoscopic colorectal cancer surgery 2006–2008

**DOI:** 10.1002/bjs.9023

**Published:** 2013-01-03

**Authors:** E F Taylor, J D Thomas, L E Whitehouse, P Quirke, D Jayne, P J Finan, D Forman, J R Wilkinson, E J A Morris

**Affiliations:** 1 Northern and Yorkshire Cancer Registry and Information Service, St James's Institute of Oncology, St James's University HospitalLeeds, UK; 2 Cancer Epidemiology Group, Leeds Institute of Molecular Medicine, University of Leeds, St James's Institute of Oncology, St James's University HospitalLeeds, UK; 3 Pathology and Tumour Biology, Leeds Institute of Molecular Medicine, St James's University HospitalLeeds, UK; 4 Translational Anaesthetic and Surgical Science, Leeds Institute of Molecular Medicine, St James's University HospitalLeeds, UK; 5 John Goligher Colorectal Unit, St James's University HospitalLeeds, UK; 6 National Colorectal Cancer Intelligence NetworkLondon, UK; 7 Cancer Information Section, International Agency for Research on CancerLyon, France

## Abstract

**Background:**

Clinical guidelines recommend that, where clinically appropriate, laparoscopic tumour resections should be available for patients with colorectal cancer. This study aimed to examine the introduction of laparoscopic surgery in the English National Health Service.

**Methods:**

Data were extracted from the National Cancer Data Repository on all patients who underwent major resection for a primary colorectal cancer diagnosed between 2006 and 2008. Laparoscopic procedures were identified from codes in the Hospital Episode Statistics and National Bowel Cancer Audit Project data in the resource. Trends in the use of laparoscopic surgery and its influence on outcomes were examined.

**Results:**

Of 58 135 resections undertaken over the study period, 10 955 (18·8 per cent) were attempted laparoscopically. This increased from 10·0 (95 per cent confidence interval (c.i.) 8·1 to 12·0) per cent in 2006 to 28·4 (25·4 to 31·4) per cent in 2008. Laparoscopic surgery was used less in patients with advanced disease (modified Dukes' stage ‘D’ *versus* A: odds ratio (OR) 0·45, 95 per cent c.i. 0·40 to 0·50), rectal tumours (OR 0·71, 0·67 to 0·75), those with more co-morbidity (Charlson score 3 or more *versus* 0: OR 0·69, 0·58 to 0·82) or presenting as an emergency (OR 0·15, 0·13 to 0·17). A total of 1652 laparoscopic procedures (15·1 per cent) were converted to open surgery. Conversion was more likely in advanced disease (modified Dukes' stage ‘D’ *versus* A: OR 1·56, 1·20 to 2·03), rectal tumours (OR 1·29, 1·14 to 1·46) and emergencies (OR 2·06, 1·54 to 2·76). Length of hospital stay (OR 0·65, 0·64 to 0·66), 30-day postoperative mortality (OR 0·55, 0·48 to 0·64) and risk of death within 1 year (hazard ratio 0·60, 0·55 to 0·65) were reduced in the laparoscopic group.

**Conclusion:**

Laparoscopic surgery was used more frequently in low-risk patients. Copyright © 2012 British Journal of Surgery Society Ltd. Published by John Wiley & Sons, Ltd.

## Introduction

Historically, the majority of colorectal cancer resections were open operations. There is growing enthusiasm for laparoscopic colorectal cancer surgery, with short-term advantages and no negative oncological consequences. Laparoscopic surgery can be challenging technically and associated with a long learning curve^1^–^3^. Although randomized trials provide evidence, their results may not always be directly transferable to the general population^1^,^4^. Monitoring the introduction and outcomes of laparoscopic colorectal cancer surgery ensures that patients receive quality care in a cost-effective manner. This study assessed the early introduction and outcomes of laparoscopic colorectal cancer surgery in the English health system.

## Methods

The National Cancer Data Repository (NCDR) contains information about every patient diagnosed with cancer in England and allows their treatment pathway to be mapped from diagnosis to cure or death. It consists of linked cancer registry, Hospital Episode Statistics (HES) and National Bowel Cancer Audit Project (NBOCAP) data.

Information was extracted from the NCDR on all individuals who underwent a major resection for primary colorectal cancer (International Classification of Diseases 10th revision C18–C20) diagnosed between 1 January 2006 and 31 December 2008. Information on age, sex, tumour site, date of diagnosis, Index of Multiple Deprivation (IMD) income category (based on postcode at diagnosis) and modified Dukes' stage at diagnosis were extracted from the registry data component of the NCDR. Modified Dukes' stage was used as, over the time period of this study, this was the only staging information captured both by English cancer registries and by the NBOCAP. Information about patient management, including operation type, approach to surgery and hospital of treatment, was derived from HES. If data on modified Dukes' stage at diagnosis or approach to surgery were missing from the HES and cancer registry data in the NCDR, this information was taken from the NBOCAP data set. Standard methods were used to identify whether each patient underwent a major resection for colorectal cancer up to 1 month before or 12 months after the date of diagnosis^5^,^6^.

Patients undergoing laparoscopic operations were identified as those with Classification of Interventions and Procedures version 4 (OPCS-4) codes indicating minimal access to abdominal cavity (Y75) or other specified approach to abdominal cavity (Y508) recorded on the same date as the major resection. Converted laparoscopic operations were identified as those with an OPCS-4 code indicating failed minimal access approach converted to open (Y714). Information on approach to surgery was also incorporated from the NBOCAP data set.

A Charlson co-morbidity score^7^ was calculated for each individual based on diagnostic codes (excluding cancer) recorded for any hospital admission in the year before diagnosis of the colorectal tumour, excluding any admission spanning the date of diagnosis. The cancer component of the Charlson index was derived from the cancer registry information in the NCDR. Patients were grouped into Charlson score categories of 0, 1, 2 and at least 3, higher scores indicating greater co-morbidity.

The urgency of surgery is known to have a strong prognostic impact on outcomes, but this information is not recorded routinely in HES. The method of admission is, however, available. Patients who were admitted as an emergency and underwent surgery within 2 days of admission were deemed to have undergone emergency surgery.

### Statistical analysis

The proportion of procedures performed via open, laparoscopic or converted surgery were examined in relation to patient age, sex, year of diagnosis, modified Dukes' stage of disease at diagnosis, tumour location, IMD category, operation type and Charlson co-morbidity score. Factors associated with the use of laparoscopic surgery were also investigated using a hierarchical random-effects binary logistic regression model, fitted using Stata® Statistical Software Release 11 (StataCorp LP, College Station, Texas, USA). The model was built with a hierarchy of patients clustered within hospitals (level 2), so allowing for correlations between patient outcomes. Co-variables (explanatory variables) in the risk-adjusted model included age (per 10-year increase), sex, tumour site, IMD income category, year of diagnosis, stage at diagnosis, Charlson co-morbidity score, operation type (elective or emergency) and operative approach. Approach to surgery was categorized as open or laparoscopic; converted operations were included in the laparoscopic group on an intention-to-treat basis. Some case-mix information (such as stage of disease and socioeconomic deprivation category) was missing from the NCDR as it was not recorded routinely in the database.

Analyses restricted to patients with complete data would not have allowed the overall outcome to be assessed. Missing data were, therefore, imputed deterministically using the ICE command^8^, with passive and substitute options and ordered logistic regression for ten imputations and ten cycles of regression switching. It was assumed that the data were ‘missing at random’. The imputation model consisted of 30-day postoperative mortality, survival time, length of hospital stay, age at diagnosis, sex, median annual workload of the hospital, modified Dukes' stage, IMD income category, operation type (elective or emergency), admission type (elective or emergency), year of diagnosis, year of operation, method of access (open, laparoscopic completed, laparoscopic converted), Charlson co-morbidity score, tumour site, hospital and cancer registry. For comparative purposes the models were built using both the imputed data set and a data set restricted to cases with complete data.

To investigate the relationship between laparoscopic treatment and the outcomes postoperative mortality, long-term survival and postoperative length of hospital stay, logistic, Cox and linear regression hierarchical random-effects models were fitted. Thirty-day postoperative mortality was defined as death within 30 days of major resection. Survival time was calculated from the date of major resection to the date of death or when censored (30 June 2010). Length of stay was defined as the number of days from major resection to the end of the associated hospital stay (calculated taking into account transfers between different hospitals). Length of stay was log-transformed before analysis with estimates back-transformed and interpreted as length of stay ratios. Length of stay values of less than 1·00 indicate a shorter stay, values greater than 1·00 indicate a longer stay, and values of 1·00 indicate no change in the duration of hospital stay due to the variable of interest.

## Results

Between 2006 and 2008, 58 135 major colorectal cancer resections were performed, of which 10 955 (18·8 per cent) were attempted laparoscopically. In total, 9303 (84·9 per cent) of these were completed laparoscopically and 1652 (15·1 per cent) were converted to open procedures. Use of the laparoscopic approach increased from 10·0 (95 per cent c.i. 8·1 to 12·0) to 28·4 (25·4 to 31·4) per cent over the study period. The proportion of patients in whom laparoscopy was attempted ranged from 0 to 65·6 per cent of the major resections in each hospital. Similarly, conversion rates varied from 0 to 46·2 per cent of all operations attempted laparoscopically. Patient characteristics are summarized in *Table* *1*, and *Table* *2* shows the results of multivariable analyses investigating the use of laparoscopic surgery.

**Table 1:** Characteristics of the study population

		Open		Laparoscopic attempted	
			
	Total	n*	Multilevel imputed†	n*	Multilevel imputed†
Age at diagnosis (years)					
< 60	10 305	8426 (81·8)	81·6 (79·1, 84·0)	1879 (18·2)	18·4 (16·0, 20·9)
60–69	16 076	12 859 (80·0)	79·4 (76·9, 81·9)	3217 (20·0)	20·6 (18·1, 23·1)
70–79	19 709	15 982 (81·1)	81·0 (78·6, 83·4)	3727 (18·9)	19·0 (16·6, 21·4)
≥ 80	12 045	9913 (82·3)	82·3 (79·9, 84·7)	2132 (17·7)	17·7 (15·3, 20·1)
Year of diagnosis					
2006	18 841	16 964 (90·0)	90·0 (88·0, 91·9)	1877 (10·0)	10·0 (8·1, 12·0)
2007	19 336	15 839 (81·9)	81·6 (78·9, 84·3)	3497 (18·1)	18·4 (15·7, 21·1)
2008	19 958	14 377 (72·0)	71·6 (68·6, 74·6)	5581 (28·0)	28·4 (25·4, 31·4)
Sex					
M	32 361	26 400 (81·6)	81·3 (78·9, 83·6)	5961 (18·4)	18·7 (16·4, 21·1)
F	25 774	20 780 (80·6)	80·6 (78·1, 83·0)	4994 (19·4	19·4 (17·0, 21·9)
Operation type					
Elective	51 530	40 865 (79·3)	79·1 (76·6, 81·7)	10 665 (20·7)	20·9 (18·3, 23·4)
Emergency	6605	6315 (95·6)	95·2 (94·2, 96·2)	290 (4·4)	4·8 (3·8, 5·8)
Modified Dukes' stage at diagnosis					
A	7583	5661 (74·7)	75·0 (72·0, 78·0)	1922 (25·3)	25·0 (22·0, 28·0)
B	20 982	16 942 (80·7)	80·4 (77·9, 82·8)	4040 (19·3)	19·6 (17·2, 22·1)
C	20 370	16 770 (82·3)	82·1 (79·8, 84·5)	3600 (17·7)	17·9 (15·5, 20·2)
‘D’	4992	4317 (86·5)	86·6 (84·6, 88·7)	675 (13·5)	13·4 (11·3, 15·4)
Unknown	4208	3490 (82·9)	–	718 (17·1)	
IMD income category					
1 (most affluent)	12 161	9753 (80·2)	79·8 (77·2, 82·4)	2408 (19·8)	20·2 (17·6, 22·8)
2	12 745	10 290 (80·7)	80·4 (78·0, 82·9)	2455 (19·3)	19·6 (17·1, 22·0)
3	12 535	10 113 (80·7)	80·8 (78·3, 83·2)	2422 (19·3)	19·2 (16·8, 21·7)
4	10 994	9019 (82·0)	81·3 (78·7, 83·8)	1975 (18·0)	18·7 (16·2, 21·3)
5 (most deprived)	8784	7298 (83·1)	81·8 (79·3, 84·4)	1486 (16·9)	18·2 (15·6, 20·7)
Unknown	916	707 (77·2)	–	209 (22·8)	–
Cancer site					
Colon	42 814	34 576 (80·8)	80·6 (78·3, 82·9)	8238 (19·2)	19·4 (17·1, 21·7)
Rectum	15 321	12 604 (82·3	81·7 (79·0, 84·5)	2717 (17·7)	18·3 (15·5, 21·0)
Charlson co-morbidity score					
0	46 957	37 717 (80·3)	80·2 (77·8, 82·0)	9240 (19·7)	19·8 (17·4, 22·2)
1	7325	6177 (84·3)	83·9 (81·6, 86·2)	1148 (15·7)	16·1 (13·8, 18·4)
2	2513	2139 (85·1)	84·9 (82·7, 87·1)	374 (14·9)	15·1 (12·9, 17·3)
≥ 3	1340	1147 (85·6)	85·4 (82·9, 87·9)	193 (14·4)	14·6 (12·1, 17·1)

Values in parentheses are *percentages and †95 per cent confidence intervals. IMD, Index of Multiple Deprivation.

**Table 2:** Odds of use of an attempted laparoscopic approach

	Complete case		Multiple imputation	
		
	Odds ratio	*P*	Odds ratio	*P*
Age at diagnosis (per 10 years)	0·99 (0·96, 1·01)	0·217	0·98 (0·96, 1·00)	0·059
Year of diagnosis	2·04 (1·97, 2·10)	< 0·001	2·06 (2·00, 2·12)	< 0·001
Sex		0·228		0·090
M	1·00	–	1·00	
F	1·03 (0·98, 1·08)		1·04 (0·99, 1·09)	
Operation type		< 0·001		< 0·001
Elective	1·00		1·00	
Emergency	0·14 (0·12, 0·16)		0·15 (0·13, 0·17)	
Modified Dukes' stage at diagnosis		< 0·001		< 0·001
A	1·00		1·00	
B	0·73 (0·68, 0·78)		0·74 (0·69, 0·79)	
C	0·66 (0·61, 0·71)		0·66 (0·62, 0·71)	
‘D’	0·43 (0·39, 0·48)		0·45 (0·40, 0·50)	
IMD income category		0·001		0·002
1 (most affluent)	1·00		1·00	
2	0·98 (0·91, 1·05)		0·96 (0·90, 1·04)	
3	0·97 (0·90, 1·04)		0·96 (0·89, 1·03)	
4	0·91 (0·84, 0·98)		0·89 (0·83, 0·97)	
5 (most deprived)	0·84 (0·77, 0·92)		0·85 (0·78, 0·93)	
Cancer site		< 0·001		< 0·001
Colon	1·00		1·00	
Rectum	0·72 (0·68, 0·76)		0·71 (0·67, 0·75)	
Charlson co-morbidity score		< 0·001		< 0·001
0	1·00		1·00	
1	0·77 (0·71, 0·83)		0·77 (0·72, 0·83)	
2	0·76 (0·67, 0·87)		0·74 (0·66, 0·84)	
≥ 3	0·68 (0·57, 0·81)		0·69 (0·58, 0·82)	

Values in parentheses are 95 per cent confidence intervals. IMD, Index of Multiple Deprivation.

Not all procedures that were attempted laparoscopically could be completed by this route and *Table* *3* describes the features of patients whose procedure was converted to an open operation. The conversion rate varied in relation to various patient factors; the multivariable analyses investigating the odds of a laparoscopically attempted operation being converted are shown in *Table* *4*. Year of diagnosis and patient age had no impact on the odds of conversion of an attempted laparoscopic procedure. The odds of conversion was reduced in women, but increased with advanced tumour stage, socioeconomic deprivation, and with rectal rather than colonic tumours.

**Table 3:** Characteristics of patients in whom laparoscopic surgery was completed and those in whom it was converted to an open procedure

	Laparoscopic completed		Converted from laparoscopic	
		
	n*	Multilevel imputed†	n*	Multilevel imputed†
Age at diagnosis (years)				
< 60	1628 (86·6)	86·5 (84·6, 88·4)	251 (13·4)	13·5 (11·6, 15·4)
60–69	2711 (84·3)	84·3 (82·6, 86·0)	506 (15·7)	15·7 (14·0, 17·4)
70–79	3133 (84·1)	83·6 (81·9, 85·3)	594 (15·9)	16·4 (14·7, 18·1)
≥ 80	1831 (85·9)	85·6 (83·7, 87·4)	301 (14·1)	14·4 (12·6, 16·3)
Year of diagnosis				
2006	1629 (86·8)	86·4 (84·3, 88·5)	248 (13·2)	13·6 (11·5, 15·7)
2007	2943 (84·2)	84·1 (82·5, 85·8)	554 (15·8)	15·9 (14·2, 17·5)
2008	4731 (84·8)	84·5 (82·9, 86·1)	850 (15·2)	15·5 (13·9, 17·1)
Sex				
M	4919 (82·5)	82·2 (80·6, 83·8)	1042 (17·5)	17·8 (16·2, 19·4)
F	4384 (87·8)	87·6 (86·3, 88·9)	610 (12·2)	12·4 (11·1, 13·7)
Operation type				
Elective	9083 (85·2)	84·9 (83·7, 86·2)	1582 (14·8)	15·1 (13·8, 16·3)
Emergency	220 (75·9)	75·6 (69·6, 81·6)	70 (24·1)	24·4 (18·4, 30·4)
Modified Dukes' stage at diagnosis				
A	1677 (87·3)	87·3 (85·3, 89·2)	245 (12·7)	12·7 (10·8, 14·7)
B	3423 (84·7)	84·5 (83·1, 86·0)	617 (15·3)	15·5 (14·0, 16·9)
C	3033 (84·3)	84·1 (82·4, 85·8)	567 (15·7)	15·9 (14·2, 17·6)
‘D’	554 (82·1)	82·4 (79·0, 85·9)	121 (17·9)	17·6 (14·1, 21·0)
Unknown	616 (85·8)	–	102 (14·2)	–
IMD income category				
1 (most affluent)	2084 (86·5)	86·3 (84·5, 88·0)	324 (13·5)	13·7 (12·0, 15·5)
2	2114 (86·1)	86·0 (84·2, 87·8)	341 (13·9)	14·0 (12·2, 15·8)
3	2057 (84·9)	85·1 (83·2, 87·0)	365 (15·1)	14·9 (13·0, 16·8)
4	1650 (83·5)	83·5 (81·7, 85·4)	325 (16·5)	16·5 (14·6, 18·3)
5 (most deprived)	1219 (82·0)	82·1 (79·6, 84·6)	267 (18·0)	17·9 (15·4, 20·4)
Unknown	179 (85·6)	–	30 (14·4)	–
Cancer site				
Colon	7062 (85·7)	85·5 (84·3, 86·7)	1176 (14·3)	14·5 (13·3, 15·7)
Rectum	2241 (82·5)	82·2 (80·2, 84·3)	476 (17·5)	17·8 (15·7, 19·8)
Charlson co-morbidity score				
0	7851 (85·0)	84·7 (83·5, 86·0)	1389 (15·0)	15·3 (14·0, 16·5)
1	968 (84·3)	84·3 (82·0, 86·6)	180 (15·7)	15·7 (13·4, 18·0)
2	316 (84·5)	84·4 (80·2, 88·6)	58 (15·5)	15·6 (11·4, 19·8)
≥ 3	168 (87·0)	87·0 (82·1, 92·0)	25 (13·0)	13·0 (8·0, 17·9)

Values in parentheses are *percentages and †95 per cent confidence intervals. IMD, Index of Multiple Deprivation.

**Table 4:** Odds of an attempted laparoscopic operation being converted to an open procedure

	Complete case		Multiple imputation	
		
	Odds ratio	*P*	Odds ratio	*P*
Age at diagnosis (per 10 years)	1·02 (0·96, 1·07)	0·553	1·04 (0·99, 1·09)	0·152
Year of diagnosis	1·05 (0·97, 1·14)	0·198	1·05 (0·98, 1·13)	0·189
Sex		< 0·001		< 0·001
M	1·00		1·00	
F	0·63 (0·56, 0·71)		0·65 (0·58, 0·73)	
Operation type		< 0·001		< 0·001
Elective	1·00		1·00	
Emergency	2·05 (1·51, 2·79)		2·06 (1·54, 2·76)	
Modified Dukes' stage at diagnosis		0·002		0·002
A	1·00		1·00	
B	1·29 (1·09, 1·52)		1·28 (1·08, 1·51)	
C	1·28 (1·08, 1·51)		1·30 (1·10, 1·54)	
‘D’	1·55 (1·20, 2·00)		1·56 (1·20, 2·03)	
IMD income category		0·001		0·001
1 (most affluent)	1·00		1·00	
2	1·03 (0·86, 1·23)		1·02 (0·86, 1·21)	
3	1·12 (0·94, 1·34)		1·12 (0·94, 1·33)	
4	1·23 (1·02, 1·48)		1·25 (1·05, 1·50)	
5 (most deprived)	1·47 (1·21, 1·80)		1·42 (1·17, 1·72)	
Cancer site		< 0·001		< 0·001
Colon	1·00		1·00	
Rectum	1·29 (1·13, 1·47)		1·29 (1·14, 1·46)	
Charlson co-morbidity score		0·720		0·755
0	1·00		1·00	
1	1·05 (0·87, 1·26)		1·04 (0·87, 1·24)	
2	1·00 (0·74, 1·36)		1·00 (0·74, 1·34)	
≥ 3	0·79 (0·49, 1·25)		0·81 (0·52, 1·25)	

Values in parentheses are 95 per cent confidence intervals. IMD, Index of Multiple Deprivation.

Analyses investigating how the approach to surgery influenced outcomes are shown in *Table* *5*. Length of stay and 30-day postoperative mortality were lower in patients in whom laparoscopic surgery was attempted. The effects were greatest among those in whom the operation was completed laparoscopically. Individuals in whom laparoscopic surgery was completed had a 40 per cent reduced risk of death within 1 year compared with those who had open surgery.

**Table 5:** Results of a multivariable regression model investigating outcomes in relation to approach to surgery

		Measure of effect	
		
	Approach to surgery	Complete case	Multiple imputation
Postoperative length of stay	Open	1·00	1·00
	Laparoscopic completed	0·65 (0·64, 0·66)	0·65 (0·64, 0·66)
	Conversion	0·92 (0·89, 0·95)	0·93 (0·89, 0·96)
30-day postoperative mortality	Open	1·00	1·00
	Laparoscopic completed	0·57 (0·49, 0·66)	0·55 (0·48, 0·64)
	Conversion	0·67 (0·50, 0·90)	0·68 (0·52, 0·90)
1-year survival	Open	1·00	1·00
	Laparoscopic completed	0·61 (0·56, 0·66)	0·60 (0·55, 0·65)
	Conversion	0·86 (0·72, 1·03)	0·84 (0·71, 1·00)

Values in parentheses are 95 per cent confidence intervals. The model was adjusted for sex, age at diagnosis, Index of Multiple Deprivation (IMD) income category, year of operation, tumour site (colon/rectum), modified Dukes' stage, operation type (elective or emergency) and presence of co-morbidity.

## Discussion

This retrospective population-based study has provided a national perspective on the adoption of laparoscopic colorectal cancer surgery and its outcomes in England. Laparoscopic surgery was attempted more frequently in patients with a better prognosis (such as elective presentation of early-stage tumours). The odds of conversion were greater in individuals with more advanced disease and those who posed a greater operative risk. Laparoscopic surgery was associated with a shorter hospital stay, a lower 30-day postoperative mortality rate and improved long-term survival.

The increased trend for laparoscopic surgery has been demonstrated in other studies^9^,^10^, but they examined only operations recorded as being completed laparoscopically and the coding of such procedures is often inaccurate in routine data sets^4^. In the present study the total number of operations attempted laparoscopically was calculated by including all procedures coded as laparoscopically converted. This approach has confirmed a rapid increase in the adoption of the techniques and provided a more complete picture.

Differences existed between the populations selected for each surgical approach, with laparoscopically treated patients tending to have elective admissions for early-stage disease. Local guidance states that minimal access surgery is not appropriate for all patients but should be available as an option under favourable conditions, for example in individuals with a body mass index (BMI) below 30 kg/m^2^, no history of major abdominal surgery, tumours category T3 or less, rectal cancers not requiring a total mesorectal excision (TME) and in the absence of clinical or radiological signs of obstruction^11^. This study has provided indirect evidence indicating that these recommendations are being implemented. These recommendations are not absolute, however, and the authors appreciate that many experienced surgeons and units routinely offer laparoscopic surgery to more complex cases (for example patients with a BMI exceeding 30 kg/m^2^ or who require TME). Unfortunately, data items that would allow identification of the more challenging laparoscopic cases (such as those with an increased BMI or advanced tumour category) are not yet available in the NCDR. Their inclusion would further increase the utility of the resource.

In the present study one in six laparoscopic procedures was converted and this proportion changed little over the course of the study. Laparoscopic experts would view this rate as too high as many units now report conversion rates below 5 per cent. This emphasizes the need for continued efforts in education and training to reduce the rate further^1^. The clinical factors that may make a conversion more likely have been documented previously^12^–^14^. The present analysis has confirmed that advanced stage of disease and co-morbidity^12^–^16^ consistently increase the likelihood of a conversion.

Randomized trials have demonstrated oncological equivalence of open and laparoscopic techniques, whereas case series have reported better outcomes in laparoscopically treated patients^17^–^19^ to the extent that one group recommended that laparoscopy should be considered routine^20^. However, patients who require conversion to open operation may have had poorer postoperative outcomes in some series^15^,^21^,^22^. The present study found that 30-day postoperative mortality, length of hospital stay and 1-year survival was better in laparoscopically treated patients (irrespective of whether a procedure was converted or not).

Patients undergoing laparoscopic surgery appeared to have a better prognosis than those receiving open surgery, although it is impossible to separate the effect of earlier disease in the laparoscopic group from any advantages arising directly from the approach. Nevertheless, appropriate selection for any surgical technique remains of paramount importance. It is apparent that some who undergo open surgery would simply not be suitable for a laparoscopic approach and it is to be expected that they have a worse prognosis. This study has highlighted some of the advantages arising from the implementation of a national programme of laparoscopic surgery for colorectal cancer. It does not conclude that laparoscopic surgery is superior to open surgery for all individuals, for which more detailed clinical information would be required.
